# Revisit of Optimal Donor Number Estimation in the Hong Kong Bone Marrow Donor Registry

**DOI:** 10.3389/fimmu.2021.638253

**Published:** 2021-04-16

**Authors:** Jenny Chung Yee Ho, Stephen Kwok Fan Cheung, Zhongyi Lui, Ivan Wing Hong Tang, Wanling Yang, Patrick Ip, Cheuk Kwong Lee, Derek Middleton, Janette Siu Yin Kwok

**Affiliations:** ^1^ Division of Transplantation and Immunogenetics, Queen Mary Hospital, Hong Kong, Hong Kong; ^2^ Department of Paediatrics and Adolescent Medicine, The University of Hong Kong, Hong Kong, Hong Kong; ^3^ Hong Kong Red Cross Blood Transfusion Services, Hong Kong, Hong Kong; ^4^ Transplant Immunology, Royal Liverpool Hospital, Liverpool, United Kingdom

**Keywords:** alleles, frequency haplotypes, HLA antigens, Chinese, matching probability

## Abstract

High resolution typing of the HLA-DPB1 locus for patient who requested for hematopoietic stem cell transplantation (HSCT) workup has recently become mandatory by the National Marrow Donor Program (NMDP) in order to facilitate matching between donors and recipients for better outcomes. The likelihood of identifying HLA matched donors in Hong Kong, on top of the existing HLA-A, -B, -C, and -DRB1 loci, is revisited in this study. HLA-A, -B, -C, -DRB1 and -DPB1 genotypes of 5,266 volunteer unrelated Chinese donors from the Hong Kong Bone Marrow Donor Registry (HKBMDR), were included in this study. Matching models were employed to determine the matching probabilities for 10/10(DPB1) and 9/10(DPB1) HLA match. The matching probabilities are 20% at 10/10(DPB1) HLA match and 55% at 9/10(DPB1) match, based on the existing 130,000 donors in the HKBMDR. The likelihoods of match become 27% and 65% respectively, by increasing the registry to 250,000. However, if DPB T-cell-epitope (TCE) model is considered in the matching, the probability will increase to 46% at 10/10 DPB1 permissive mismatching. Our findings provide vital information about the future planning on the targeted recruitment size, HLA typing and search strategies of the donor registry and arose the transplant physicians’ acceptability to 9/10(DBP1) or 10/10(DBP1) HLA match. Nevertheless, the marrow donor registry has planned for increasing the registry size and bringing down the age of recruited donors which will ultimately enhance patient outcome.

## Introduction

The detrimental graft-versus-host disease (GVHD) remains a major challenge after curative hematopoietic stem cell transplantation (HSCT). Systemic outcome analysis has shown that HLA-DPB1 mismatch had resulted in increased risk of acute GVHD. Transplantation with non-permissive DPB1 mismatch was shown to be associated with higher transplant-related mortality (1). Starting from 27 February 2021, HLA typing of DBP1 loci has become mandatory for patients requesting for HSCT workup from the National Marrow Donor Program (NMDP). In light of better outcome for HSCT, optimal matching between donors and recipients are recommended at high resolution in the HLA-A, -B, -C, -DRB1 and -DPB1 loci. Due to the population-specific allelic variation and the extremely high level of HLA gene polymorphism, the availability of optimal HLA-matched unrelated donors and cord-blood units has always been a concern (2, 3). As a result, donors with mismatched HLA antigens may also be considered in many situations. However, these HLA mismatches may lead to an 8% reduction per loci in the 5-year overall survival rate after HSCT (4). The additional information on DPB1 loci may help clinician on final donor selection by reviewing the matching at DPB1 to enhance the patient outcome when more than one potential donors are available for HSCT.

Volunteer unrelated donor database has been managed by the Hong Kong Bone Marrow Donor Registry (HKBMDR). At present, there are close to 130,000 stem cell donors in HKBMDR and 38 million donors in the Bone Marrow Donors Worldwide (BMDW) (5). Continual growth on the number of donors has been achieved globally. However, it accompanied with significant resource implication in donor recruitment and HLA typing. Therefore, strategic donor recruitment becomes very important account of the donor registry planning. Many crucial factors, including recruitment on more young male donors (6) or focus on the recruitment of donors with rare human leukocyte antigen (HLA) phenotypes (7), donors from ethnic minority (8–11), and recruitment activities based on HLA frequency differences at regional priority setting (12–15).

Estimation of matching probability, including mixed patient population, provides vital information for donor recruitment strategy planning and framework for international stem cell donor exchange (16). We have used the calculations based on HLA-A, -B, -C and -DRB1 loci high-resolution haplotype frequencies (HF) of our own population to estimate the donor pool size earlier (17).

The linkage disequilibrium between HLA-DPB1 and other loci are weak due to a hot-spot of recombination between HLA-DPB1 and HLA-DQB1 loci (18). A big proportion of unrelated donor HSCTs were performed across HLA-DPB1 mismatches (19, 20). HLA-DPB1 alloantigens are target of graft-versus-leukemia (GVL) or graft-versus-host (GVH) disease mediated by alloreactive T cells (21–24). However, only 3-57% of HLA-DPB1 were typed in the HLA DNA typed unrelated donors from varies registries (25). Since it was well known that racial and ethnic background play a profound role in adult-donor availability and match probabilities (26), the same phenomenon was proven in our previous study (17). We estimated the donor pool and matching probability on HLA 10/10(DBP1) matching with reference to our recent publication on the gene and HF of the Hong Kong population (27). To our knowledge, this is the first study to revisit the calculation of matching probabilities of our population and the estimation of donor size based on the additional DPB1 requirement.

## Materials and Methods

### Sample Collection and Genotyping

The gene frequency and HF as reported previously were used in the analysis (27). In brief, Next generation sequencing supplemented with sequence-specific primer was used to define allele combinations and some specific alleles with 5,266 donors. HF was calculated from these results using Markov Chain Monte Carlo (MCMC) algorithm PHASE (28). Matching model was then utilized by using the calculated HF and effective adult-donor registry size for each group, with the assumption of genotypes in Hardy-Weinberg equilibrium (HWE) (29, 30).

HLA-DPB1 typing was assigned based on T-cell Epitope algorithms version 2.0 assignment (https://raw.githubusercontent.com/ANHIG/IMGTHLA/Latest/tce/dpb_tce.csv) and also the online tool at https://www.ebi.ac.uk/ipd/imgt/hla/dpb_v2.html (31). The TCE group assignment was reported for all HLA-DPB1 alleles according to the Release 3.38.0 of the IPD-IMGT/HLA Database, released 2019-10. The predicted immunogenicity of the HLA-DPB1 matching will be presented as Permissive, Non-Permissive GvH or Non-Permissive host-versus-graft (HvG).

### Statistics Analysis

The frequencies of HLA-A, -B, -C, -DRB1 and -DPB1 alleles were calculated from the number of observed genotypes. MCMC simulation from Guo and Thompson was utilized to assess the Hardy-Weinberg equilibrium for each loci *via* PHASE (32), and the deviance of genotype frequency within each loci was detected by PHASE invoking Arlequin (33). *P* value of <0.01 was considered to be statistically significant.

Formulae described by Schmidt et al. has been utilized in this study with modification (16). In brief, the probability *p(n)* for any patient from their own population to identify at least one matched donor in a registry including n individuals of a donor population is given *p(n)* =Ʃ_i_ *f*_i_[1-(1-*f*_i_)^n^] with *p(n)* being the matching probability in “*n*” sample size, *fi* being the frequencies of the *i*-th genotype and *i*-th is any genotype from the rank of genotype frequencies in the order from the highest to the lowest in a donor population. The estimated HF was used to derive the genotype frequencies under the assumption of HWE.

## Results and Discussion

Data from the recently published HLA genotype and haplotype frequencies of the HKBMDR (27) was applied in this study. Characteristics of these HLA haplotypes in Hong Kong were summarized in **Table 1**.

**Table 1:** Characteristics of the haplotypes of Hong Kong.

HLA loci available		A-C-B-DRB1-DPB1
Sample size (N)		5,266
Sample size (2N)		10,532
Number of haplotypes (>0.006%)		3,326
Sum (%) of haplotype frequencies within the top	10	10.7
	25	16.0
	50	21.1
	100	28.1
	250	40.2
	500	51.7
	1,000	65.6
Number of haplotypes with frequency	≥0.01	3
	≥0.005	9
	≥0.001	126
	≥0.0005	323
	≥0.0001	1,918

In concordance with our previous study (17), it was found that the number of haplotypes was significantly increased with number of donor samples. This increase is exclusive for our local population, as a plateau of number of haplotypes with increase in sample size was not observed in other ethnic groups, e.g. Caucasians and European populations (34). Mori et al. reported that a significant higher level of the occurrence of common haplotypes (0.01%) was observed in Asian Americans than in Caucasian Americans in the NMDP database. This suggested that the Caucasian Americans had a smaller degree of genetic diversity than Asian Americans (35). Similar findings from a large sample database that the occurrence of common haplotypes in Asian or Pacific Islanders (API) was also higher than Caucasians (34). However, whether the same phenomenon will be observed when HLA-DPB1 is considered requires further elucidation.

**Figure 1:**
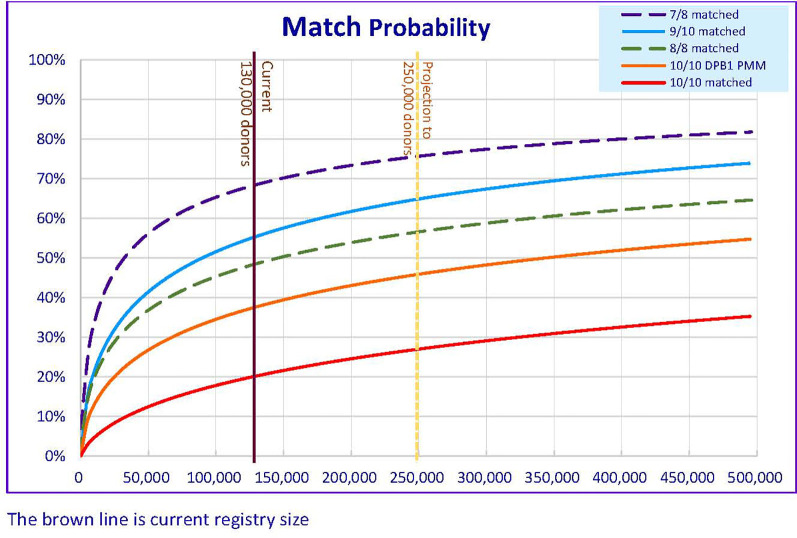
Matching Probability (MP) of varies level of HLA match against different donor registry size in HKBMDR. DPB1 PMM – with HLA-DPB1 permissive mismatch.

A similar methodology was applied in calculating the likelihood of finding a “matched” donor in US (26), likelihood of finding an 8/8 HLA match or ≥ 7/8 HLA match by different donor registry size in Hong Kong was reported in previous study for matching A, B, C, DRB1 loci only (17). With the increase in the number of donors in the HKBMDR to 130,000 as of December 2020, the likelihood of finding an available 8/8 HLA matched donor is 49% and 69% for finding 7/8 HLA matched donor (**Figure 1**). The results were comparable to those figures found among Asians, Pacific Islanders, and Native Americans (26). However, when taking into account of matching for HLA-DPB1 loci, the likelihood of finding an available 10/10(DBP1) HLA matched donor is 20% while 55% for finding 9/10(DBP1) HLA matched donor. Similar finding was observed in a Finnish retrospective study in which only 32.6% of local donors or 19.3% of both local and foreign donors were HLA-DPB1 matched with HSCT patients (36). In our data, the matching probability increases to 38% when taking into account of the DPB1 T-cell-epitope (TCE) permissive mismatching model.

TCE Groups has been utilized in classifying HLA-DPB1 mismatches that might be tolerated (permissive) or would increase risks (non-permissive) after unrelated HSCT. If HLA‐DPB1 matching with TCE Groups is considered, beneficial effect during donor selection has been shown in various studies (37, 38). Donors with a permissive HLA-DPB1 group are preferred over those showing a non‐permissive HLA-DPB1 group, among those 9/10(DPB1) and 10/10(DBP1) potential donors. “DPB1 TCE3 grading” has been implanted in OptiMatch with the evident published by Zino et al. (39, 40). The new score was assigned based on the 3 TCE Groups algorithm according to the T cell cross-reactivity patterns (31).

IPD‐IMGT/HLA website provided the original feature of DPB1 TCE3 algorithm and have been used in the BMDW Search & Match Service. The discrimination of permissive or non‐permissive HLA‐DPB1 mismatches is determined based on whether the donor and patient alleles belong to the same (permissive) or different (non‐permissive) TCE Groups. There are total of 81 combinations of the HLA-DPB1 typing resulting for TCE version 2 assignment (**Supplementary Table 1**).

Greater heterogeneity in HLA typing of the Hong Kong Chinese was found where compared with other populations (34, 41, 42). Therefore, to enhance the chance of successful donor search, a larger donor pool is warranted. In concordance with the findings by Dehn and Buck, the likelihood of matching in HLA-A, -B, -C, -DRB1 and -DQB1 10 alleles for Asian Americans was also inferior than Caucasian Americans for 7/8 or 9/10(DQB1) matched unrelated donor search was also lower (98% vs 88%) (43–45).

In addition to the matching issue, attrition of donors due to age and contact unavailability may pose another negative impact on the likelihood of finding a donor. Based on the previous registry size of 100,000, the attrition rate was 2% or 2,000 per year. As shown in the projection (**Figure 1**), increasing the registry size to 250,000 in five-year time, 26,600 new recruitments per year is required to achieve matching likelihood at 46% for 10/10(DBP1) HLA-DPB1 permissible Match or 65% for ≥ 9/10(DBP1) HLA Match. An annual recruitment of 26,600 is a big rise compared to the current of 8,000 per year. Extra resources should be sought to cover the cost in donor recruitment and HLA typing. A survey was conducted to identify the crucial factors that affect the motivation of stem cell donation in Hong Kong (46). To enhance the recruitment ratio of the younger age group, recruitment program targeting a specific age group, especially for student at higher education may facilitate better recruitment rate and longer maintenance for donation to maximize the cost-effectiveness. Targeted educational activities such as Stem Cell Donation campaign, including educational talks to students and parents, promotion video on social media and social networking platforms and roadshows may help to enhance the recruitment of youngsters.

Racial and ethnic background in a donor registry has been reported to affect the adult-donor availability (26). The current analysis has not taken into the account of adult-donor availability which may have substantially lower match likelihoods. In addition, donors from the patient’s own racial and ethnic group has shown to have the highest matching probability (47), this probability may also be enhanced if donors from other racial and ethnic groups could be available. Registry with donors that have a relatively low occurrence of inter-racial or inter-ethnic marriage might have less chance to have donors identified from other groups. The overall donor available rate is less than 30% (27) and it will expect to be lowered when additional loci is considered.

In the above estimation, the matching probability from around 3 million Chinese donors registered in China and Taiwan registries has not taken into account, which may provide extra donor matching. Furthermore, the matching probability of the cord blood units which are readily available and require less stringent HLA matching was not included in this calculation. Cord blood would be used as an alternative when adult donor is not readily available in many transplant centers. The issue of relatively low stem cell dose for adult size recipient has been resolved by the application of double cord blood units, and has been proven success clinically (48, 49). Whether cord blood can eventually substitute the need of a large registry is still debatable.

Although only 5,266 donors HLA haplotype frequencies have been included in the current study, some rare alleles may not be covered in the presence analysis and affect the accuracy of the estimation. Nonetheless, common haplotype for those with frequencies above 0.2% should be covered. The information provided in this study provided an overview of the matching probability for the local population and facilitate the formulation of donor recruitment target and planning for extra resources in order to support the cost in donor recruitment and HLA typing. Establishment of a cost-effective bone marrow donor registry with an expanded donor pool is utmost important to enhance the likelihood of matching, shorten donor search time in the same ethnicity as domestic donors are more likely to donate stem cells (47). Moreover, it circumvents the shipment restriction or border control especially during the COVID-19 pandemic. This will facilitate timely HSCT in order to catch the best timing during patient remission period, and thus enhance the success rate of HSCT and patient outcome. A more comprehensive model of analysis for inclusion of availability of donor, incomplete or discrepant donor typing and loss of contact would be desired. With the continuation of donor HLA typing by the NGS technology, a revisit of the analysis with a larger sample size would be warranted in the future in order to obtain a more accurate estimation to cover the rare HLA alleles.

## Data Availability Statement

The raw data supporting the conclusions of this article will be made available by the authors, without undue reservation.

## Ethics Statement

Ethical review and approval was not required for the study on human participants in accordance with the local legislation and institutional requirements. The donors have provided their written informed consent to perform HLA typing for HKBMDR.

## Author Contributions

The study was designed by WY and JK. Data was collected by JH, SC, IT, CKL and JK. The computation and statistical analyses were performed by IT, ZL, WY and JK. The samples were provided by CKL and JK. The manuscript was written by JH, SC, IT, PI, CKL, DM and JK. All authors contributed to the article and approved the submitted version.

## Conflict of Interest

The authors declare that the research was conducted in the absence of any commercial or financial relationships that could be construed as a potential conflict of interest.

## Supplementary Material

Supplementary Table 1 81 Combinations of DPB1 TCE v2 assignment and matching outcome.
