# Supplementary material for: Revisit of Optimal Donor Number Estimation in the Hong Kong Bone Marrow Donor Registry
Source: Front Immunol. 2021 Apr 16;12:638253. doi: 10.3389/fimmu.2021.638253 (PMC8085527; doi:10.3389/fimmu.2021.638253)
Supplement: Supplementary Table 1 — 81 Combinations of DPB1 TCE v2 assignment and matching outcome. [file Table_1.pdf]

Supplementary Table 1.

	P1	P2	D1	D2	DPB1 TCE matching
1	1	1	1	1	Permissible
2	1	1	1	2	Permissible
3	1	1	1	3	Permissible
4	1	1	2	1	Permissible
5	1	1	2	2	Non-Permissible GvH
6	1	1	2	3	Non-Permissible GvH
7	1	1	3	1	Permissible
8	1	1	3	2	Non-Permissible GvH
9	1	1	3	3	Non-Permissible GvH
10	1	2	1	1	Permissible
11	1	2	1	2	Permissible
12	1	2	1	3	Permissible
13	1	2	2	1	Permissible
14	1	2	2	2	Non-Permissible GvH
15	1	2	2	3	Non-Permissible GvH
16	1	2	3	1	Permissible
17	1	2	3	2	Non-Permissible GvH
18	1	2	3	3	Non-Permissible GvH
19	1	3	1	1	Permissible
20	1	3	1	2	Permissible
21	1	3	1	3	Permissible
22	1	3	2	1	Permissible
23	1	3	2	2	Non-Permissible GvH
24	1	3	2	3	Non-Permissible GvH
25	1	3	3	1	Permissible
26	1	3	3	2	Non-Permissible GvH
27	1	3	3	3	Non-Permissible GvH
28	2	1	1	1	Permissible
29	2	1	1	2	Permissible
30	2	1	1	3	Permissible
31	2	1	2	1	Permissible
32	2	1	2	2	Non-Permissible GvH
33	2	1	2	3	Non-Permissible GvH
34	2	1	3	1	Permissible
35	2	1	3	2	Non-Permissible GvH
36	2	1	3	3	Non-Permissible GvH
37	2	2	1	1	Non-Permissible HvG
38	2	2	1	2	Non-Permissible HvG
39	2	2	1	3	Non-Permissible HvG
40	2	2	2	1	Non-Permissible HvG
41	2	2	2	2	Permissible
42	2	2	2	3	Permissible
43	2	2	3	1	Non-Permissible HvG
44	2	2	3	2	Permissible
45	2	2	3	3	Non-Permissible GvH
46	2	3	1	1	Non-Permissible HvG
47	2	3	1	2	Non-Permissible HvG
48	2	3	1	3	Non-Permissible HvG
49	2	3	2	1	Non-Permissible HvG
50	2	3	2	2	Permissible
51	2	3	2	3	Permissible
52	2	3	3	1	Non-Permissible HvG
53	2	3	3	2	Permissible
54	2	3	3	3	Non-Permissible GvH
55	3	1	1	1	Permissible
56	3	1	1	2	Permissible
57	3	1	1	3	Permissible
58	3	1	2	1	Permissible
59	3	1	2	2	Non-Permissible GvH
60	3	1	2	3	Non-Permissible GvH
61	3	1	3	1	Permissible
62	3	1	3	2	Non-Permissible GvH
63	3	1	3	3	Non-Permissible GvH
64	3	2	1	1	Non-Permissible HvG
65	3	2	1	2	Non-Permissible HvG
66	3	2	1	3	Non-Permissible HvG
67	3	2	2	1	Non-Permissible HvG
68	3	2	2	2	Permissible
69	3	2	2	3	Permissible
70	3	2	3	1	Non-Permissible HvG
71	3	2	3	2	Permissible
72	3	2	3	3	Non-Permissible GvH
73	3	3	1	1	Non-Permissible HvG
74	3	3	1	2	Non-Permissible HvG
75	3	3	1	3	Non-Permissible HvG
76	3	3	2	1	Non-Permissible HvG
77	3	3	2	2	Non-Permissible HvG
78	3	3	2	3	Non-Permissible HvG
79	3	3	3	1	Non-Permissible HvG
80	3	3	3	2	Non-Permissible HvG
81	3	3	3	3	Permissible

Abbreviations: TCE = T cell epitope; P = patient; D = donor; HvG = host versus graft; GvH = graft versus host;
